# A Huge PVDF Adsorption Difference Between Resveratrol and ε-Viniferin Allows to Quantitatively Purify Them and to Assess Their Anti-Tyrosinase Property

**DOI:** 10.1007/s10337-014-2707-8

**Published:** 2014-06-15

**Authors:** Cécile Morel-Salmi, Audrey Julia, Claire Vigor, Joseph Vercauteren

**Affiliations:** 1 Faculty of Pharmacy, Laboratory of Pharmacognosy, Caudalie R&D, 15 Av. Charles Flahault, 34093 Montpellier, France; 2 Faculty of Pharmacy, Laboratory of Pharmacognosy, Institute of Biomolecules Max Mousseron (IBMM) (UMR 5247-CNRS-UM1-UM2-ENSCM), 15 Av. Charles Flahault, 34093 Montpellier, France

**Keywords:** Polyvinylidene fluoride (PVDF), *trans*-Resveratrol, *trans*-Epsilon-viniferin, *Vitis vinifera*, Tyrosinase inhibition

## Abstract

Repeated chromatographic analyses of polyphenolic vine stalks extracts allowed us to note a huge adsorption difference on polyvinylidene fluoride (PVDF), between *trans*-resveratrol **1** and (+)-*trans*-ε-viniferin **2**. We could optimize the conditions (solvent, saturation of the process), for this polymer to adsorb very selectively **2**, with regard to the monomer **1** that remains in solution. Since membrane filters made of PVDF are quite often used for HPLC samples filtration, this observation prompted us to inform phytochemists studying plant stilbenoid contents. Based on this background information, we developed a straightforward and inexpensive enrichment process for either **1** and/or **2**, from crude *Vitis vinifera* stalks extracts, allowing to get them in a pure form. Having at hand large amounts of these two pure compounds, they were tested and compared to a set of other relevant molecules for some biological properties: *trans*-ε-viniferin **2** was shown to be the most powerful tyrosinase inhibitor, among all samples tested.

## Introduction

For more than two decades now, there is a constant and increasing interest in obtaining bulk amounts of natural polyphenolics from the stilbenoid series. Namely, in the recent years, hundreds of papers report the major biological properties of resveratrol. This monomer building block of the series is described, among others, as a powerful antioxidant [1], anti-cancer [2] and anti-inflammatory [3] agent. Not only resveratrol itself, but more recently, some of its derivatives (monomers and oligomers) were shown to have interesting pharmacological properties. This is the case, for instance, of the inhibitory effects on tyrosinase activity of oxyresveratrol [4].

**Fig. 1:**
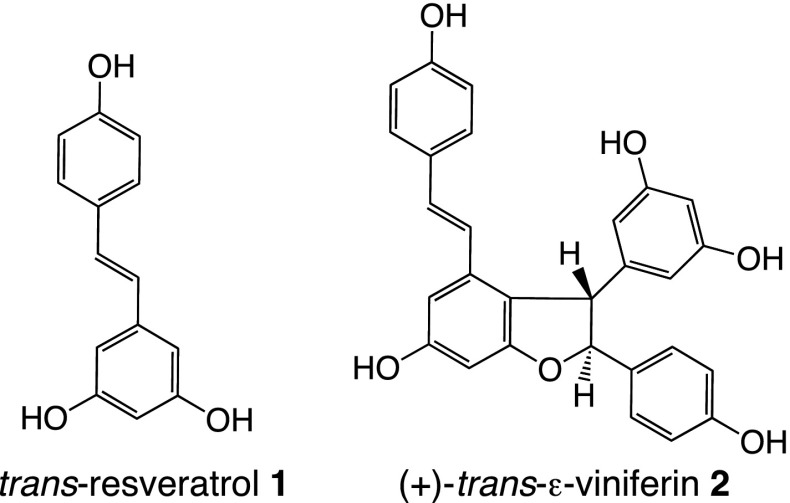
Chemical structures of *trans*-resveratrol **1** and *trans*-ε-viniferin **2**

Indeed, since back to the 1990s, the “wooden” part of vine is known by us to be one of the most valuable natural sources of stilbenoids: the two major occurring constituents being the monomer *trans*-resveratrol **1**, and one of its dimers, (+)-*trans*-ε-viniferin **2** (Fig. 1).

In order to identify the richest stalks from various red and white grape varieties (cépages), we prepared specific polyphenolic extracts. Chromatographic dosages (HPLC analysis) of the many crude extract solutions allowed us to observe an unprecedented phenomenon: for instance, in reiterated analysis of one same extract, **2** was detected in either more than 20 % yields or totally absent (0 %)! Trying to understand such unpredictable results, we could evidence that these discrepencies originated from the type of the syringe filter used to filtrate the analyzed samples.

We then wondered if this behaviour, crucial at a very small scale (filtration of very diluted solutions), could be transposed to “gram scale” of crude *Vitis vinifera* stalks extracts and could lead to preparative amounts of enriched fractions or of pure either **1** or **2**. The large amounts of these two stilbenoids provided by this process would give us the occasion to test their biological activities, namely, as tyrosinase inhibitors.

## Experimental

### Chemicals and Reagents

The polyvinylidene fluoride (PVDF) is a commercial polymer (Alfa Aesar, A Johnson Mattley Company, Ward Hill, MA), with a density = 0.38 and is used as it is. *trans*-Resveratrol **1** [or (*E*)-5-(4-hydroxystyryl)benzene-1,3-diol] and (+)-*trans*-ε-viniferin **2** {or 5-[(2*S*, 3*S*)-6-hydroxy-2-(4-hydroxyphenyl)-4-(4-hydroxystyryl)-2,3-dihydrobenzofuran-3-yl]benzene-1,3-diol} obtained through the described process were authenticated by HPLC chromatograms comparison with homemade authentic sample references and of their ^1^H NMR (500 MHz) spectra. Mushroom tyrosinase (E.C. 1.14.18.1, T3824 Sigma, 25,000 units mg^−1^), l-DOPA (L0400000 Fluka, crystalline), kojic acid (K3125 Sigma, crystalline 100 % purity), ascorbic acid (A1300000 Fluka, crystalline 100 % purity), arbutin (A4256 Sigma, ≥98 % purity) and mushroom tyrosinase were purchased from Sigma-Aldrich, Saint-Quentin Fallavier France. Acetone, ethanol, acetonitrile (HPLC grade) and ethyl acetate are from Labover, Montpellier, France. All solvents were rectified prior to be used.

### HPLC Analyses

HPLC analyses of the extracts were performed using a liquid chromatograph Model 2695 from Waters (Guyancourt, France) connected to a dual absorbance detector (Model 2487), monitored at 330 nm, and equipped with a Synergi 4 μm hydro RP 80A column (250 × 2.0 mm I.D., 4 μm particle size, Phenomenex, Le Pecq, France) and with a guard cartridge of the same material. The mobile phase was composed of two solvents: solvent A = water with 0.015 % H_3_PO_4_ (v/v) and solvent B = CH_3_CN, at a flow rate of 0.2 mL min^−1^ with the following gradient: 32–50 % B (0–20 min), 50 % B (20–25 min), 50–100 % B (25–29 min) and 100 % B (29–45 min). Each 2 mL oligomers of resveratrol (OR) sample (0.25 mg mL^−1^) solution in the same solvent as the starting gradient (solvent A = 68 % and solvent B = 32 %; v/v), was filtrated through a disposable syringe filter, pore size 0.45 µm from Macherey–Nagel (Hoerd, France), either of PET-type (Chromafil^®^ Xtra PET-45/25-Polyester), or of PVDF-type (Chromafil^®^ P-45/25-PVDF), prior to be injected (10 μL) from the autosampler. Amounts of **1** and **2** in each injected sample were calculated by using individual calibration curves for peaks at retention times of 9.2 and 13.5 min, for **1** and **2**, respectively.

### Enrichment Process

Preparation of crude stilbenoid extracts (oligomers of resveratrol = OR): OR extracts are obtained from finely powdered stalks of *V. vinifera*, following the process described in [5]. Vine shoots, harvested every year during winter pruning, are selected according to their richness in stilbenoids (highest concentrations are found in different “cépages”, depending on the year). In this study, crude OR extract from “Merlot noir” stalks (ORMn) used to set up the conditions for the enrichment process, contained initially 12.4 % of **1** and 15.8 % of **2** (w/w).

Preparation of the PVDF support: The PVDF (500 g = 1.3 L) is stirred in a 5 L flask, for 2 h at room temperature, with 2 L of acetone–water (4:96; v/v = the chosen solvent A used to set up the conditions for the enrichment process), in order to moisten it. The thus “prepared support” is considered to be “ready to use”, after removal of the excess of solvent by filtration.

Adsorption step = obtention of the *trans*-resveratrol **1** enriched fraction: The solution of 10 g of ORMn in 1 L of the chosen solvent A is added to the flask containing the 500 g prepared PVDF. The resulting slurry is stirred for 15 min, before to be filtered. The support is rinsed with 1 L of chosen solvent A, in three times. The different filtrates are pooled and concentrated by evaporation in vacuo at room temperature of the organic phase. The residual aqueous solution is freeze-dried to yield 5.7 g of fraction 1. Analyzed by HPLC, without any filtration, it was shown to contain 736 mg of **1** and 407.8 mg of **2**.

Desorption step = obtention of the **2** enriched fraction: The support is then shaken for 15 min with 1 L of the solvent B chosen to set up the conditions for the enrichment process (acetone–water 80:20; v/v), before filtration. This operation was repeated once. The two filtrates are pooled and concentrated by evaporation in vacuo to get rid of the organic phase. The resulting aqueous solution is freeze-dried to furnish the fraction 2 (2.56 g): analyzed by HPLC, without preliminary filtration, it was shown to contain 142 mg of **1** and 645 mg of **2**.

### Measurement of Tyrosinase Inhibition Activity

Conditions used: The conditions used to measure the capacity of each compound to inhibit tyrosinase l-DOPA oxidase activity were adapted from Kang [6]. 40 μL of phosphate buffer (=control; 67 mM, pH 6.8) or of solutions of **1** (25, 50, 125, 250 and 500 μM), **2** (6.5, 13, 26, 52 and 104 μM), kojic acid (12.7, 25, 51, 127 and 254 μM), arbutin (63, 127, 254, 507 and 1014 μM), or ascorbic acid (100, 200, 400, 1000 and 2000 μM) in phosphate buffer are added to 40 μL of mushroom tyrosinase (248 U/mL) and incubated at 37 °C for 30 min in a 96-well microplate. Then, 40 μL l-DOPA (2.5 mM) and 80 μL phosphate buffer were added to the solution. Optical density (OD) of each assay, relative to the control, was measured at 492 nm by using a microplate reader (Tecan^®^ Infinite M200 spectrophotometer).

Results expression: Inhibition of enzyme activity by each sample is expressed as  % inhibition compared to the control and represented by the following equation: [1 − (OD_sample_ × OD_control_^−1^)] × 100.

## Results and Discussion

### Observation of an Unexpected Phenomenon

**Fig. 2:**
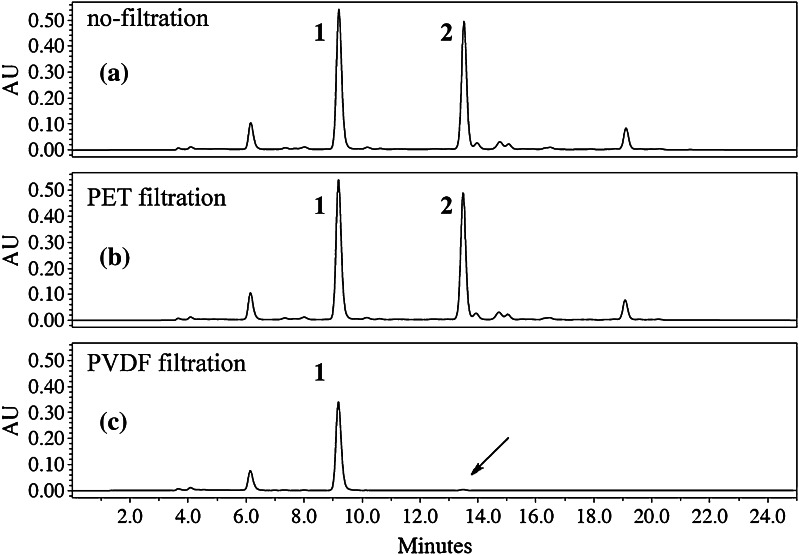
Chromatographic profiles of the same crude stalk stilbenoid extract (OR) measured at 330 nm: **a** without filtration, **b** filtration on a PET filter, and **c** filtration on a PVDF filter

Looking for the red or white grape varieties (cépages) with the highest stilbenoids content, we were dosing **1** and **2** by HPLC–UV. 10 μL of the solution (0.25 mg mL^−1^) of each stalk extract [5], in the mixture of 0.015 % H_3_PO_4_ (v/v) in water and 32 % CH_3_CN (v/v), was injected in triplicates, on the column after filtration on a disposable syringe filter. In such conditions, concentrations of **2** were ranging from 25 to 0 %. We were fully confident with the results, because there was a good repeatability between the triplicates (SD mean ≤0.1 %), until a major difference appeared between two measurements of the same extract: the two first values were speaking for 16.9 ± 0.06 % of **2**, and 13.4 ± 0.01 % of **1**, while the third one for 0 % of **2**, and 13.5 % of **1** (Fig. 2).

Rapidly, we found that this huge difference was due to the membrane type of the syringe filter used to “prepare” the solutions before injection: the two first values (16.9 ± 0.06 % of **2**) were obtained when using a polyethylene terephthalate (PET) filter (Fig. 2b), while the last one (0 % of **2**, *arrow* Fig. 2c) resulted from a polyvinylidene fluoride (PVDF) filter.

### Recommendation to the Phytochemists

It is of general practice to pass sample solutions through filters, for HPLC analysis. While previous reports have already mentioned that, depending on the nature of the membrane polymer, adsorption of polyphenols, in general, is a major issue for fouling the filters during microfiltration of wine [7] and even, specifically, that PVDF filters should not be used with resveratrol and its monomeric derivatives since they are adsorbed onto [8], it is the first time here that such a difference of adsorption on PVDF is observed between the monomer **1** and one of its dimer **2**. Thus, it is of prime importance to let phytochemists involved in the study of plant metabolites of the stilbenoid series know that such a filter would not allow to get to their true composition. The only way to ensure that the filter has no impact on the analyses is to run an analysis without filtration (Fig. 2a). To have reliable dosages of stilbenoids, PVDF membrane filters must be avoided. Possibly, this could have led to erroneous results in the past, as it was most probably the case, in the Mazza’s study: these authors precised the use of PVDF membrane filters before HPLC injections [9], aiming at assessing the amounts of **1** and **2** contained in grape cane waste. Also, because PVDF, chemically “inert”, durable and biocompatible, is widely used in food and medicinal applications for ultra- or micro-filtration [10], this information should be extended to those fields.

### Enrichment Process

An ORMn extract, containing initially 12.4 % of **1** and 15.8 % of **2** (w/w) was used to set up the conditions for the enrichment process. The PVDF capacity to adsorb **2** during filtration of sample solutions to be analyzed by HPLC, was clearly shown to be limited: the peak of **2** at 13.5 min in the chromatogram, after having disappeared completely, is growing up, then, depending on the concentration or on the volume of the filtered solution. This capacity was measured first and the w/w ratio optimized to be able to treat “grams” of crude extracts: 500 g of PVDF for 10 g of ORMn extract containing 1.58 g of **2**. The enrichment process includes at least one cycle of adsorption–desorption onto the PVDF support.

Adsorption step: The much more efficient adsorption of **2** compared to **1** onto the perfluorinated polymer has something to see with the peculiar dimeric structure. The solvent for this step (solvent A) must be chosen to be capable to solubilize all the constituents of the OR extracts, but the minimum of **2** in the presence of PVDF. This solvent is—in particular—a monophasic mixture of water and acetonitrile, acetone or methanol, in which the water represents from 99.8 % to at least 70 % (v/v). In the present case, we have used 1 L of acetone–water (4:96; v/v). Adsorption is facilitated by moistening the PVDF support, in the presence of the solvent A. The resulting slurry is stirred for 15 min, to allow selective adsorption of **2** onto the PVDF. During this first step of the process, **2** is adsorbed onto the PVDF, while **1** mainly keeps in solution. The slurry is filtrated and the support is rinsed two times by the same solvent A. The different filtrates are pooled prior to be concentrated by evaporation in vacuo of the organic part. The residual aqueous solution is freeze-dried to yield fraction 1, enriched in **1**.

Desorption step: Then, *trans*-ε-viniferin **2** is desorbed from the support by an ideal solvent, to obtain a solution enriched in **2**. To insure an efficient desorption of **2** from the support, the elution solvent (solvent B) must also be a monophasic mixture of water and an organic solvent, but containing now, a larger proportion of the latter. It is, for example, a mixture of acetonitrile–water (95:5; v/v), acetone–water (90:10; v/v) or methanol–water (50:50; v/v). In the present case, we have used acetone–water (80:20; v/v). This step is realized by stirring the adsorbed-support with the chosen solvent B, followed by filtration. This desorption step is repeated twice, and the two fractions are combined prior to the evaporation under reduced pressure of the organic solvent. The resulting concentrated aqueous solution is freeze-dried to give the fraction 2, enriched in (+)-*trans*-ε-viniferin **2**.

**Fig. 3:**
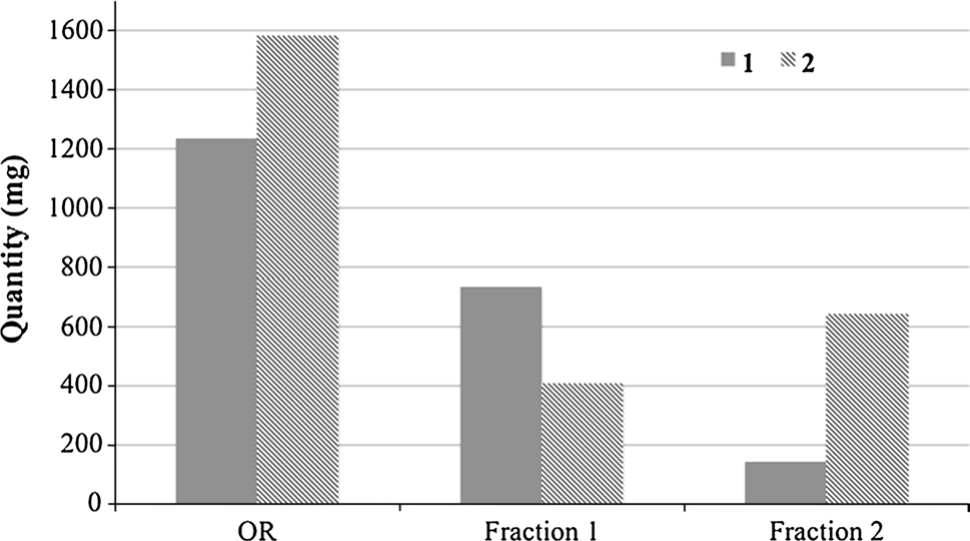
Enrichments in **1** and **2** obtained with one cycle of the process from ORMn extract

Enrichment data: By submitting 10 g of the ORMn extract with a **1** to **2** starting ratio of 0.78 and a **2** to **1** ratio of 1.28 (all the proportions of **1** and **2** being expressed in *w*/*w*), to one cycle of this process, in the conditions described in this section, we have obtained 5.7 g of a fraction 1 (12.9 % in **1** and 7.2 % in **2**) with a **1**–**2** ratio of 1.79, enriched 2.3 times in **1**, and 2.56 g of a fraction 2 (5.5 % in **1** and 25.1 % in **2**) with a **2**–**1** ratio of 4.56, enriched 3.6 times in **2** (Fig. 3).

By repeating this process to a suitable enriched fraction, it is possible to enrich further the fraction in either **1** or **2**. Unfortunately, each cycle of this very fast and easy process is accompanied by an important loss of material: at least 29 % (w/w) in **1**, and 33.6 % (w/w) in **2**.

Comparison with other process: Several processes have already described [11, 12] the separation of *trans*-resveratrol and *trans*-ε-viniferin from crude extracts. Our process relies on the observation that a big difference of adsorption on PVDF exists between monomer **1** and one of its dimer **2**. To the best of our knowledge, it is the first time that PVDF “powder” is used as a support to enrich fractions in either ones of those compounds from *V. vinifera* stalk extracts [13].

Tyrosinase inhibition effect: Having at hands pure **1** and pure **2**, we examined some of their biological properties. In this study, we report on the tyrosinase inhibitory effects of these two compounds, with respect to the second step of tyrosinase oxidation [14] (l-DOPA oxidase). The most potent **2**, was then compared to other references such as arbutin, kojic or ascorbic acids, frequently used for cosmetic purpose as anti-tyrosinase (Table 1).

**Table 1:** Summary of the tyrosinase inhibition of compounds **1**, **2** and of other known compounds

	IC_50_ (μM)	Activity ratio against **2**
*trans*-ε-Viniferin **2**	4.1 ± 0.5	1
Kojic acid	16.9 ± 1.1	1/4.1
*trans*-Resveratrol **1**	52.8 ± 1.4	1/12.9
Arbutin	55.1 ± 8.9	1/13.4
Ascorbic acid	255 ± 10	1/62.2

IC_50_ values represent the concentration of sample causing 50 % inhibition of tyrosinase

Each value represents the mean ± SD of three experiments

Mushroom tyrosinase was chosen to run these experiments, as it is classically used to detect and develop tyrosinase inhibitors [15]. The present study established that kojic acid inhibits the enzyme very effectively (IC_50_ = 16.9 μM). However, compound **2** clearly revealed to be the most active tyrosinase inhibitor ever tested by us, with an IC_50_ = 4.1 μM (Table 1). Thus, (+)-*trans*-ε-viniferin **2** is more than four times more potent than kojic acid (IC_50_ = 16.9 μM), and 62-fold more active than ascorbic acid (IC_50_ = 255 μM) to inhibit tyrosinase (Table 1). *trans*-Resveratrol **1** has a moderate inhibitory activity (IC_50_ = 52.8 μM), quite similar to arbutin (IC_50_ = 55.1 μM).

## Conclusions

Because PVDF polymer was shown to adsorb selectively and very efficiently **2** from a solution, while **1** mainly keeps in solution, phytochemists using HPLC as an analytical tool, should keep in mind that the choice of the membrane filter for samples preparation is crucial to get reliable results, at least with such stilbenoids. Taking advantage of this information, and because PVDF polymer is very stable, abundant, and cheap, we developed a simple, inexpensive and a preparative scale purification process of **1** and of **2** from the crude extracts of *V. vinifera* stalks. As a pure compound, **2** can therefore be easily sterilized and becomes potentially a useful active ingredient to treat melanosis or most of the other melanin overproduction disorders (melanoma), as well as a skin-lightening agent (against dark spots in dermatocosmetics, for instance).
